# Beyond accuracy: leveraging ASSURED criteria for field evaluation of point-of-care tests for food animal diseases

**DOI:** 10.3389/fvets.2023.1239111

**Published:** 2023-08-31

**Authors:** Sylvester Ochwo, Andres M. Perez, María Sol Pérez Aguirreburualde

**Affiliations:** Center for Animal Health and Food Safety, College of Veterinary Medicine, University of Minnesota, Saint Paul, MN, United States

**Keywords:** point-of-care tests, field evaluation, modified framework, FIT-REASSURED, animal health

## Abstract

The growing availability of point-of-care tests (POCTs) for food-animal diseases offers opportunities for timely diagnosis, facilitating the efficient implementation of control measures. However, field assessment of new POCTs are yet to be standardized. This paper discusses the opportunity of expanding the current approach for the evaluation and validation of POCTs in food animal disease diagnosis, highlighting the limitations of traditional practice that primarily relies on estimating diagnostic accuracy (sensitivity and specificity). Here, the use of a protocol referred to as FIT-REASSURED, a modified framework combining the ASSURED and REASSURED criteria, is proposed to comprehensively assess POCTs. FIT-REASSURED encompasses key criteria such as fitness for purpose, real-time connectivity, ease of specimen collection, affordability, sensitivity, specificity, user-friendliness, rapidity and robustness, equipment-free operation, and deliverability. By incorporating these attributes, FIT-REASSURED provides a customizable approach to assess the accuracy, affordability, and utility of POCTs. Through collaborative efforts among stakeholders, the implementation of a standardized scorecard based on these FIT-REASSURED criteria can improve the reliability and practicality of POCTs in food-animal health.

## Introduction

Diagnostic tests are essential in both clinical and field settings. In clinical settings, these tests are used to determine the need for treatment or to make decisions regarding the appropriate level of treatment. In field settings, they are employed to assess the frequency of diseases, identify their causes, and often select animals for necessary actions like culling (1). These tests play a crucial role in enabling informed decisions on disease management (prevention and control), and the safe trade of animals and their products (2). Furthermore, in the context of notifiable diseases, these tests support the efforts of veterinary services in implementing national control plans by facilitating timely and confident decision-making when there is a suspicion of disease, and minimizing the transmission of diseases and their impact on animal populations and public health (3).

Traditionally, diagnostic testing is laboratory-based, often involves expensive equipment, and requires skilled personnel to operate. While laboratory-based testing is known for its reliability and accuracy, it faces cost, sample transportation, and result turnaround time challenges (4–6). In settings with limited access to veterinary services or in remote areas, conducting laboratory-based disease diagnosis becomes particularly difficult due to factors such as the unavailability of laboratory facilities, equipment and technical personnel; economic and logistical constraints associated with testing costs and sample transportation, as well as the need for timely decision-making. Consequently, point-of-care tests (POCTs) have emerged as a practical alternative in this kind of scenario, as they bring the appealing advantage of enabling on-site testing that can be performed by individuals with limited technical expertise, providing rapid results at a lower cost compared to laboratory tests (5).

Technology advancements, portable equipment versions and the availability of thermal stable reagents, primarily used in human POCTs, have further facilitated their adoption in veterinary diagnostics. Such progress, along with the growing global demand for animal products and the need to manage emerging and re-emerging animal diseases, has intensified the demand for POCTs in the veterinary sector. The market for POCTs in veterinary diagnostics is expected to be worth 5.6 billion US dollars by 2030, with a current growth rate of 12.3% (7). Demand for POCTs in food animals will account for about 40% of this expected market value (7).

However, as the demand for point-of-care testing in food animals continues to grow, it becomes crucial to establish harmonized guidelines and criteria to evaluate the suitability of commercially available POCTs for field use. This need becomes even more significant due to the fact that these tests are often administered by individuals with varying levels of expertise in diagnostic testing. Standardized guidelines are crucial in ensuring the effectiveness and reliability of these tests across different settings and user proficiency levels. Additionally, priorities for POCT operational needs, such as portability of equipment, power source, ease of use, availability of controls, and cost of the tests differ across settings. Therefore, the development of harmonized criteria would ensure the appropriate selection of POCTs for their specific purposes, improving their overall efficiency. Furthermore, these guidelines would serve as a valuable resource for diagnostic test manufacturers when designing and validating test device performance prior market release, and for official veterinary services when making informed decisions about suitable tests.

As a precedent, significant progress has been made in the development of guidelines for reporting the accuracy of diagnostic tests, such as the “Standards for Reporting of Diagnostic Accuracy Studies (STARD)” statement (8, 9). STARD aims to enhance the completeness and transparency in reporting diagnostic accuracy studies by, providing a standardized list of essential items. Discussions have also taken place regarding the adaptation of STARD for animal disease studies and use of a different instrument (QUADAS) for assessing methodological quality (10). Additionally, the “Standards for Reporting of Diagnostic Accuracy Studies that use Bayesian Latent Class Models (STARD-BLCM)” have been developed for situations where a reference standard is absent (11). These standardized checklists are valuable tools that focus on diagnostic accuracy and can be applied to report various infectious disease diagnostic test results, including laboratory tests and POCTs.

In this article, we propose using the ASSURED criteria (12, 13), which stands for Affordable, Sensitive, Specific, User-friendly, Rapid and Robust, Equipment-free, and Deliverable, as well as its modified version, REASSURED (14, 15), as comprehensive guidelines for assessing POCTs for infectious diseases of food animals. Originally recommended by the World Health Organization (WHO) (12), these criteria encompass attributes beyond diagnostic accuracy, offering a valuable opportunity for comprehensive evaluation of POCT attributes, through the integration with other valuable pre-existing tools such as STARD. This approach enhances the assessment, validation, and selection of suitable POCTs for specific epidemiological settings, thereby improving disease control and management strategies while enabling better resource utilization by procuring the most appropriate POCTs for their specific needs.

## Why is it relevant to standardize and harmonize criteria for the assessment of POCTs operational performance for food animal diseases?

The increasing use of POCTs in food animals without proper assessment of their operational field performance raises concerns about their true ability to effectively deliver reliable diagnoses. The operational performance of a diagnostic test encompasses factors beyond accuracy alone, including precision, speed, ease of use, cost-effectiveness, robustness, and suitability for the intended purpose or specific context/population. Studies by Hobbs et al. (5) and Velayudhan and Naikare (16) have discussed these challenges extensively and highlighted issues such as inconsistent and unclear validation data from test manufacturers, limitations in field validation and evaluation studies, difficulties in validating tests for specific pathogens, and the lack of guidelines for ensuring quality control in veterinary POCTs.

As further evidence supporting the points mentioned above, when evaluating commercially available tests for African swine fever (ASF) and foot-and-mouth disease (FMD), two of the most significant transboundary animal diseases, it becomes apparent that the majority of these commercial tests have been primarily assessed under laboratory conditions (17) (Table 1).

**Table 1:** List of commercially available POCTs for two of the world’s most important transboundary animal diseases, ASF and FMD.

Commercial test (Manufacturer)	Type of test	Disease tested for	Analyte and sample type tested	Peer reviewed publications
ASFV Ag Rapid Test Kit (RingBio)	Lateral flow test	ASF	Antigen, serum, plasma, whole blood	(18)
INgezim® ASF CROM Ag (Ingenasa)	Lateral flow test	ASF	Antigen, whole blood	(19)
ASF rapid antigen test card (Shenzhen)	Lateral flow test	ASF	Antigen, whole blood	(20)
PenCheck (Silverlake research)	Dipstick test	ASF	Antigen, whole blood	Not yet available
Rapid ASFV Ag (Bionote)	Lateral flow test	ASF	Antigen, serum, plasma, whole blood	Not yet available
Excelsior Biosystem Sentinel® ASF Virus Antibody Rapid Test	Lateral flow test	ASF	Antibodies, serum, plasma, whole blood	Not yet available
Herdscreen® ASFV Ab test (GlobalDX and Algenex)	Lateral flow test	ASF	Antibodies, plasma, whole blood	Not yet available
INgezim® ASFV-CSFV CROM Ab (Ingenasa)	Lateral flow test	ASF/CSFV	Antibodies, serum, whole blood	(21)
INgezim® PPA CROM (Ingenasa)	Lateral flow test	ASF	Antibodies, serum, whole blood	(22)
Genesig q16 qPCR (Genesig)	Portable PCR	ASF	DNA, whole blood, serum	Not yet available
Indical IndiField portable (Biomeme)	Portable PCR	ASF	DNA, whole blood, serum, tissues	(23)
T-CORE8 (Tetracore)	Portable PCR	ASF	DNA, whole blood, tissues	(24)
Genie III (OptiGene)	LAMP Assay	ASF	DNA, serum, swabs	(25)
POCKIT Central (GeneReach)	Portable PCR	ASF	DNA, serum, tissues, whole blood	(26)
T-COR TM 8 (Tetracore)	Portable RT-PCR	FMDV	RNA, oral swabs, epithelial tissue, esophageal–pharyngeal (OP) fluid, serum	(27)
Rapid FMD NSP Ab (Bionote)	Lateral flow test	FMDV	Antibodies, serum, plasma, whole blood	Not yet available
FMD Type A antigen rapid test card (Shenzhen)	Lateral flow test	FMDV	Antigen, serum, whole blood	Not yet available
FMD Type O antigen rapid test card (Shenzhen)	Lateral flow test	FMDV	Antigen, serum, whole blood	Not yet available
FMD antigen rapid test card for bovine, goat, and porcine (Shenzhen)	Lateral flow test	FMDV	Antigen, feces, saliva, vesicles	Not yet available
Swine foot-and-mouth disease virus antibody rapid test (Shenzhen)	Lateral flow test	FMDV	Antibodies, serum, whole blood	Not yet available
Herdscreen® FMD NSP Ab	Lateral flow test	FMDV	Antibodies, plasma, whole blood	Not yet available
Herdscreen® FMD NSP Ab (Swine)	Lateral flow test	FMDV	Antibodies, plasma, whole blood	Not yet available
SVANODIP® FMDV-Ag (Svanova)	Lateral flow test	FMDV	Antigen, vesicular fluid, epithelial suspension	(28)
VDRG® FMDV 3Diff/PAN Ag Rapid kit (Median diagnostics Inc.)	Lateral flow test	FMDV	Antigen, epithelial tissue, blisters, ruptured lesions	Not yet available

The data presented in Table 1 reveals that although the current WOAH’s terrestrial manual incorporates criteria such as fitness for purpose, analytical and diagnostic accuracy, repeatability, and reproducibility in its guidelines for validating diagnostic tests (3, 12), there is a gap between these guidelines and their implementation in practice. Furthermore, reports in the WOAH journal emphasize the importance of attributes like ruggedness and robustness in validating POCTs (15, 16). This highlights the need for the development of an explicit framework for assessing the operational performance of POCTs in real-world settings. Such a framework would bridge the gap and ensure that the guidelines and considerations outlined by WOAH are effectively applied in practice to enhance the reliability and effectiveness of POCTs. Although the establishment of legislation for the use of POCTs falls within the purview of the veterinary authority in each member country rather than WOAH, employing standardized criteria to evaluate and validate the performance of POCTs under field conditions will greatly contribute to providing valuable data and evidence to guide decision-making and legislation development in countries where such regulatory frameworks are lacking.

## The World Health Organization’s ASSURED criteria and its modifications

In 2002, the World Health Organization (WHO) and the Special Programme for Research and Training in Tropical Diseases (TDR) recognized the crucial importance of innovative, effective, and affordable diagnostic methods for sexually transmitted diseases in humans, especially in developing countries. A set of criteria known as ASSURED was established to outline the desired qualities for new POCTs. These criteria include affordability, sensitivity, specificity, user-friendliness, rapid and reliable performance, equipment-free operation, and deliverability to those in need (12). The ASSURED criteria have played a pivotal role in guiding the development, validation, and regulation of POCTs for human healthcare over the last two decades. However, with the advent of wireless technology and the realization that individuals without specialized training may be conducting these tests, a revised version of the criteria, known as REASSURED, has been proposed. This updated standard places a greater emphasis on facilitating uncomplicated sample collection and connectivity in real-time (14, 15).

While originally designed for POCTs in human health, the fundamental characteristics that led to the development of the ASSURED criteria - accessibility, affordability, and accuracy - are equally critical for point-of-care testing in animal health. Except for the unique idea of fitness for purpose, which addresses the special diagnostic requirements for animal health, particularly in farm or field settings, the core ideas are transferable across both domains.

## Suggesting the use of fitness for purpose (FIT-REASSURED) criteria for POCTs for food animal diseases

We propose the use of FIT-REASSURED, an adapted framework that combines the concept of fitness for purpose with the REASSURED criteria, to assess performance suitability of POCTs for infectious food animal diseases. It offers a comprehensive and flexible framework for assessing the performance and usability of POCTs. In the following sections, we outline the attributes of FIT-REASSURED criteria as they pertain to animal health. To illustrate its application, we conducted a preliminary assessment of five commercially available POCTs for ASF using FIT-REASSURED criteria, highlighting their strengths and weaknesses in Table 2.

**Table 2:** Assessment of commercial POCTs for ASF using the FIT-REASSURED framework, highlighting how each test meets the fitness for purpose criteria and the other attributes of the framework.

Commercial POCT	Fitness for purpose	Real-time connectivity	Ease of specimen collection	Affordability*	Sensitivity*	Specificity*	User-friendliness	Rapidity and robustness	Equipment-free setup or simplicity	Deliverable
INgezim ASF CROM Ag (Ingenasa)	Evaluated with samples from experimentally infected pigs and with samples from ASF field cases in domestic pigs and wild boars	Not incorporated into the design of the test but can be adapted using a mobile phone app	Requires blood collection	US$ 5.80–10.45 per test	68–76% compared to rt-PCR	96% compared to rt-PCR	Requires minimal training	Results in 15 min Kit has a shelf life of 12 months at 2–8°C Excellent diagnostic agreement when compared to Antigen detection ELISA	No equipment is needed Produces minimal waste but could be infectious	Can be purchased in bulk but requires storage at 2–8°C
ASF rapid antigen test card (Shenzhen)	Evaluated in a laboratory using field samples	Not incorporated into the design of the test but can be adapted using a mobile phone app	Requires blood collection	US$3.5 per test	65%	76%	Requires minimal training	Results in 15 to 20 min Not robust, Kit should be stored in the dark away from sunlight and direct blows	No equipment is needed Produces minimal waste but could be infectious	Can be purchased in bulk but requires special storage conditions
PenCheck (Silverlake research)	Evaluated in laboratory	Not incorporated into the design of the test but can be adapted using a mobile phone app	Requires blood collection and dilution	US$2.5 per test	−90% reported by the manufacturer −27% reported by an independent laboratory	−100% reported by the manufacturer −92% reported by an independent laboratory	Requires minimal training	Results in 20 min Not much information available about storage and, shelf life	No equipment is needed Produces minimal waste but could be infectious	Can be purchased in bulk but not yet licensed for use in the united states
INgezim® PPA CROM (Ingenasa)	Evaluated both in the laboratory and in the field -Purpose of field evaluation was for sero-surveillance	Not incorporated into the design	Requires blood collection	US$5 per test	82% under field conditions in wild boar, compared to immunoperoxidase monolayer Assay −99% under laboratory conditions when compared with ELISA	96% under field conditions with wild boar samples, compared to immunoperoxidase monolayer Assay −99.9% under laboratory conditions when compared with ELISA	Requires minimal training	Results in 10 min Reagents are stable between 4–25°C	No equipment is needed -Produces minimal waste but could be infectious	Can be purchased in bulk and stored at room temperature
Indical IndiField (Indical and Biomeme)	Evaluated for early detection of ASF in the field	Has cloud storage and real-time connectivity to mobile phone	Requires blood collection	High equipment cost US$10,000 -US$5–10 per sample extraction	High, up to 100%	High, up to 100%	Requires Highly trained staff	Results in 90 min DNA extraction can be done manually PCR reagents are lyophilized and stable under field conditions	Uses a portable PCR machine and manual nucleic acid extractor/cartridge	Can be purchased in bulk but the sale of reagents is restricted in some parts of the world

^*^Estimates of costs of kits, sensitivity, and specificity were obtained from “The WOAH ASF Reference Laboratory Network’s overview of African swine fever diagnostic tests for field application.”

### Fitness for purpose

The concept of fitness for purpose pertains to the validation of a test, ensuring its performance characteristics for a specific intended use and under well-defined conditions (29). WOAH has identified major purposes that encompass demonstrating disease freedom in animal populations, confirming the absence of infection or agents in individual animals or products for trade purposes, assessing the effectiveness of eradication policies, confirming the clinical diagnosis of suspected cases, estimating infection prevalence for risk analysis, and determining immune status after vaccination, among others (29, 30). However, it is crucial that test validation be conducted with a clear definition of the intended purpose. For instance, a specific purpose could be to detect antigens against the African Swine Fever (ASF) virus in domestic pig herds presenting with hemorrhagic fever following an outbreak in an ASF-free geographical area. The purpose in this example would be to ensure that a POCT is validated for the detection of active cases of ASF at the herd level. Such a test could have low diagnostic sensitivity but high specificity at the individual sample level, and still be used to achieve this purpose by selecting pigs with clinical signs of hemorrhagic fever and increasing the number of samples what would increase sensitivity of the test at herd level. However, it is crucial to recognize that this approach may not be appropriate in other scenarios, for example, for certifying the health status of individual animals in a quarantine station.

In accordance with the concept of fitness for purpose, establishing the cutoff for diagnostic sensitivity of a POCT used at the herd level involves considering the cost associated with failing to detect a positive case (false negative). Given the potential risk of further disease spread resulting from a false-negative result, the diagnostic sensitivity cutoff is determined to minimize the possibility of missing infected animals. This decision-making process takes into account the purpose of the test, as well as the trade-offs between accuracy, operational attributes, and costs. By carefully considering these factors, a selected POCT can effectively support disease prevention efforts.

### Real-time connectivity

Is the capability of systems, networks, or devices to create and maintain continuous, immediate, and synchronized communication or data exchange. It would be desirable for a POCT device to connect with external devices or systems like mobile phones or data networks. This allows for the real-time transmission and exchange of test results, enabling long-distance remote monitoring and collaboration among veterinarians, researchers, regulatory bodies, and other relevant stakeholders.

### Ease of specimen collection

The simplicity and convenience with which samples from animals may be obtained for diagnostic testing. Tests that require less invasive or non-invasive sampling techniques and are easy to conduct are highly desirable, particularly in farm or field environments where access to specialized equipment or trained personnel may be limited. For example, a test that utilizes saliva or oral fluids obtained from a rope hung in a pen is preferred over one that necessitates blood collection.

### Affordability

Inexpensive and cost-effective POCTs must be easily available and feasible to use without causing financial strain. Affordable POCTs promote widespread use and improved disease detection and management, especially in resource-limited settings. This leads to better animal health outcomes and more effective disease control.

### Sensitivity

The ability of a diagnostic test to correctly identify animals that are truly infected with a specific pathogen or disease. This relates to the accurate classification of animals as being diseased, lowering the possibility of sick animals going undetected and spreading disease.

### Specificity

The ability of a diagnostic test to correctly identify animals that are truly free from a specific pathogen or disease. Specificity is a critical characteristic since it ensures that healthy animals are not mistakenly classified as infected, eliminating wasteful treatments and consequent interruptions of trade or animal movement.

### User-friendliness

The test’s ease of use and intuitive design render it accessible to a wide range of users, including producers, para-veterinarians, and individuals without professional experience. Clear instructions, straightforward processes, and minimal steps ensure that users can confidently execute and interpret the test. The test process must be simple, necessitating minimal handling of samples or reagents and requiring no additional laboratory materials.

### Rapidity and robustness

A rapid POCT offers quick turnaround, producing data in a short period, typically minutes to hours, enabling prompt decision-making and the implementation of suitable actions. A robust POCT is made to withstand difficult field conditions, changes in sample quality, and environmental variables without losing effectiveness (30).

### Simplicity and environmental friendliness

Tests that do not rely little on complex laboratory equipment and take into account the effects of their use and the waste they generate on the environment. A POCT should not produce toxic waste in large quantities that can contaminate grazing grounds and water for humans and animals.

### Deliverability to end-users

Making tests and reagents accessible to farmers, technicians, and veterinarians requires practical considerations like supply chain management, packaging, storage, and distribution networks. By addressing these factors, we can ensure that essential POCTs are readily available to those who need them.

## Discussion and conclusion

The use of POCTs to support laboratory diagnosis of food animal diseases presents a great opportunity to strengthen disease prevention and control by increasing the accessibility to fast, cost-effective and reliable results. To fully leverage this opportunity, steps need to be taken to ensure that POCTs that reach the market are of the desired quality, enabling veterinary services to select the most appropriate tests for their needs. However, the challenge lies in the observation that approaches for validating diagnostic tests, including POCTs, primarily focus on laboratory-based assessment (29). This approach overlooks the impact of environmental factors in the field and other attributes (affordability, operational attributes) which affect test performance and usability. These factors significantly influence the diagnostic accuracy and usability of POCTs, making it unreliable to extrapolate test results validated in one setting to another. Furthermore, the trade-offs between diagnostic accuracy, operational attributes, and the associated costs vary in different scenarios or settings.

To address these challenges, initial suggestions have been made to assess attributes such as test robustness and ruggedness during field validation of POCTs. Furthermore, there has been a proposal to incorporate a specific stage in WOAH guidelines, which would focus on the field validation of POCTs (30, 31). In addition, the design of checklists and scorecards for evaluating POCTs and other diagnostic tests has already been suggested (32, 33). However, what is still missing is a comprehensive framework that can be integrated with existing initiatives such as STARD and STARD-BLCM. To bridge this gap, we propose the FIT-REASSURED framework. This framework aims to integrate ongoing efforts and establish a systematic approach for assessing the suitability of POCTs for food animal diseases. While STARD emphasizes methodological rigor and the reporting of diagnostic accuracy studies, FIT-REASSURED takes into account practical applicability factors alongside diagnostic accuracy.

For example, it is widely recognized that different animal populations infected with the same pathogen exhibit diverse epidemiological characteristics. This variation underscores the need for tailored approaches in disease management. While most POCTs for food animal diseases provide simple dichotomous results, there are also tests that offer more detailed quantitative or semi-quantitative assessments, providing a deeper understanding of disease status (16, 34, 35). The availability of continuous results presents a valuable opportunity for veterinary services to validate different cut-off values based on animal species and sample type, thereby improving specificity and accuracy in disease diagnosis and management. This further emphasizes the importance of integrating and promoting the application of harmonized guidelines, such as STARD, for reporting animal disease studies.

Using the FIT-REASSURED framework as a guideline, stakeholders involved in the development and implementation of POCTs could systematically assess and prioritize different attributes within each criterion of the framework, considering their impact on the performance of a POCT for a given purpose. As demonstrated in Table 2, documenting this information allows for a reduction of the number of POCTs to be validated, streamlining the overall evaluation process. However, it is important to note that the necessary information to make informed decisions is currently partially available in the inserts and protocols. The systematic implementation of the FIT-REASSURED framework aims to address this gap and make it a goal for POCT kit manufacturers to comprehensively provide this information in the future.

The application of the FIT-REASSURED framework to assess ASF POCTs shown in Table 2 proved helpful in identifying the strengths and weaknesses of each test. Common positive aspects across all the POCTs evaluated were their ability to offer rapid results and the requirement for minimal training of users. The major challenges identified were the need for field evaluation of the tests for a predetermined purpose; all the POCTs required blood as a sample, which requires a trained person for proper sample collection; and the need for cold storage of reagents, which may affect the test’s robustness and deliverability.

Moving forward, once the fitness for purpose has been established and a shortlist of POCTs has been created, it is crucial to prioritize and weigh the remaining REASSURED attributes based on user needs. Engaging experts in diagnostics and epidemiology is essential to effectively assessing the relative importance of these attributes in the context of POCTs. Expert elicitation tools can be utilized to gather insights and opinions, allowing decision-makers to derive weights for each attribute based on their perceived significance. This process enables informed prioritization of attributes that align with the specific needs and requirements of the diagnostic tool.

We anticipate collaboration among various stakeholders, including researchers, regulatory organizations, veterinary services, and test manufacturers, to drive the advancement of FIT-REASSURED. Researchers will contribute to developing and testing tools for ranking and scoring the attributes of FIT-REASSURED, while incorporating Bayesian Latent Class models to measure diagnostic accuracy (36) and test repeatability. Economic studies will assess cost–benefit analysis and compare POCTs with laboratory tests to evaluate cost-effectiveness. Regulatory bodies will review the data generated by researchers and test manufacturers to develop appropriate guidelines for the use of POCTs. Manufacturers should consider FIT-REASSURED attributes when developing new POCTs to ensure desired quality standards are met. Veterinary services can utilize the framework to select and assess the suitability of commercially available POCTs, fostering collaboration and driving improvement. Collectively, these efforts will enhance the reliability and practicality of POCTs in managing food animal diseases.^1^

## Data availability statement

The original contributions presented in the study are included in the article/supplementary material, further inquiries can be directed to the corresponding author/s.

## Author contributions

SO conceptualized the idea for this perspective article, reviewed the available literature, and drafted the manuscript. AP and MA refined the idea, reviewed, and edited the manuscript. All authors contributed to the article and approved the final version for submission.

## Funding

This study was funded by the United States Department of Agriculture-Animal and Plant Health Inspection Service (USDA APHIS) through a grant (grant number AP22VSD&B000C028) for “Improving national capacity for early detection of foreign animal disease incursions: critical evaluation of field accuracy, standard of procedures, risks, challenges, and opportunities for the implementation of point of care platforms for National Animal Health Laboratory Network (NAHLN).”

## Conflict of interest

The authors declare that the research was conducted in the absence of any commercial or financial relationships that could be construed as a potential conflict of interest.

## Publisher’s note

All claims expressed in this article are solely those of the authors and do not necessarily represent those of their affiliated organizations, or those of the publisher, the editors and the reviewers. Any product that may be evaluated in this article, or claim that may be made by its manufacturer, is not guaranteed or endorsed by the publisher.

## Footnotes

^1^K. Inui, C. Gallardo, R. Portugal, L. Dixon, C. Baton, D. Williams, et al., The OIE ASF Reference Laboratory Network’s overview of African swine fever diagnostic tests for field application.
